# BAIAP2L2 is a novel prognostic biomarker related to migration and invasion of HCC and associated with cuprotosis

**DOI:** 10.1038/s41598-023-35420-0

**Published:** 2023-05-29

**Authors:** Hui Wei, Jing Yang, Xia Chen, Mengxiao Liu, Huiyun Zhang, Weiming Sun, Yuping Wang, Yongning Zhou

**Affiliations:** 1 The First Clinical Medical College, Lanzhou University, Lanzhou, 730000 China; 2 Department of Gastroenterology, Key Laboratory for Gastrointestinal Diseases of Gansu Province, The First Hospital of Lanzhou University, Lanzhou, 730000 Gansu Province China; 3 Key Laboratory for Gastrointestinal Diseases of Gansu Province, The First Hospital of Lanzhou University, Lanzhou, 730000 China; 4 Department of Endocrinology, The First Hospital of Lanzhou University, Lanzhou, 730000 China

**Keywords:** Cancer genomics, Tumour biomarkers, Computational biology and bioinformatics

## Abstract

Hepatocellular carcinoma (HCC) is a leading cause of cancer-related death worldwide, and its pathophysiological mechanisms remain unknown. IRSp53 family members, such as BAIAP2L1, participate in the progression of multiple tumors. However, the role of BAIAP2L2 in HCC remains unclear. This study comprehensively analyzed the potential role of BAIAP2L2 in HCC using bioinformatic techniques. The expression of BAIAP2L2 in HCC was analyzed using The Cancer Genome Atlas (TCGA), Gene Expression Omnibus (GEO), International Cancer Genome Consortium (ICGC), and Human Protein Atlas (HPA) databases and in vitro experiments. In addition, the prognostic value of BAIAP2L2 in HCC was analyzed using the TCGA database. TCGA and GEO database were used to analyze the role of BAIAP2L2 in immune features. We also explored the function of BAIAP2L2 in methylation and cuprotosis. The CellMiner database was used to analyze the relationship between BAIAP2L2 expression and drug sensitivity. Our study revealed that BAIAP2L2 is overexpressed in HCC and promotes the migration and invasion of HCC cells. BAIAP2L2 may affect the prognosis of HCC by regulating immunity, methylation, and cuprotosis. BAIAP2L2 is a novel HCC prognostic gene involved in immune infiltration associated with cuprotosis and may be a potential prognosis and therapeutic target for HCC.

## Introduction

Hepatocellular carcinoma (HCC) is ranked as the fourth leading cause of cancer-related death worldwide^1,2^. In contrast to the declining disease burden of many other cancers, the overall burden of HCC worldwide has increased progressively over time. The age at HCC onset varies significantly across different regions of the world^3^. Chronic hepatitis B and hepatitis C infections are the most important causes of HCC, accounting for approximately 80% of cases worldwide^4,5^. As society continues to progress and evolve, nonalcoholic fatty liver disease (NAFLD), a representative metabolic liver disease, has become the leading cause of HCC growth in the United States, the United Kingdom, and France in recent years. One study reported that NAFLD was associated with a 2.6-fold increased risk in HCC^6^. The incidence of NAFLD-associated HCC is expected to increase substantially worldwide by 2030^7^. Currently, serum alpha-fetoprotein (AFP) remains the most broadly used biomarker for HCC screening, early diagnosis, and evaluation of treatment efficacy and prognosis^8^. Nevertheless, not all HCCs secrete AFP, and AFP could be elevated in cases of cirrhosis or hepatitis^9^. Therefore, there is an urgent need to explore HCC markers.

We identified BAIAP2L2 as a gene that can affect HCC prognosis by taking the intersection of HCC prognostic genes from the TCGA database, differentially expressed genes (DEGs) from the GEO database (GSE39791) and I-BAR family genes (Supplementary Fig. S1A). BAIAP2L2, a member of the IRSp53 family, was localized to rab13-positive vesicles and intercellular junctions of the plasma membrane. BAIAP2L2 has the typical BAR structural domain of family members, which does not induce membrane protrusion or invagination but promotes the formation of planar membrane sheets^10^. In recent years, an increasing number of studies have suggested that BAIAP2L2 is strongly associated with the development of various cancers. For example, BAIAP2L2 is a promising diagnostic and prognostic biomarker for prostate and lung cancers^11–14^. BAIAP2L2 promotes gastric cancer cell proliferation and metastasis by activating the AKT/mTOR and Wnt3a/β-catenin pathways^15^. In addition, it has been suggested that BAIAP2L2 may serve as a prognostic marker for patients with non-small cell lung cancer with low expression of PD-1 and EGFR^16^. BAIAP2L2 expression is significantly elevated in HCC, indicating its potential as a biomarker for predicting the recurrence of HCC^17^.

Tumor immune barrier determines the efficacy of immunotherapy in the HCC microenvironment^18^. Tumor-infiltrating immune cells within the tumor microenvironment can predict the prognosis of cancer patients^19^. It has been shown that BAIAP2L2 may have immunological value in HCC^20^. Previous studies have identified a crucial role of cuprotosis in tumor prognosis and tumor immune microenvironment^21,22^. However, there are unknown that some associations of cuprotosis-mediated genes with BAIAP2L2 in HCC. In addition, DNA methylation patterns are strongly associated with human diseases, including cancer^23,24^. Therefore, this study explored the expression level and prognostic value of BAIAP2 in HCC and investigated the effects of BAIAP2L2 on tumor immune infiltration, methylation, cuprotosis and drug sensitivity. Ultimately, the scratch assay and transwel assay confirmed that BAIAP2L2 affected the migration and invasion of HCC cells. This study might provide innovative insights to further clarify the pathological mechanism of HCC and identify novel prognostic and therapeutic targets.

## Materials and methods

### Gene expression analysis

RNA-seq data were downloaded and converted to TPM format and log2-transformed from level 3 HTSeq-FPKM format in the TCGA–LIHC project. The Wilcoxon test was used to compare the differences in BAIAP2L2 expression between HCC tissues and normal tissues. R software^25^ and R packages^26^ were used for statistical and visual analyses of the data. In addition, differences in BAIAP2L2 transcription levels were verified using the GEO (GSE39791)^27,28^and ICGC (LC-RIKEN, JP)^29^ databases. Immunohistochemistry (IHC) images of BAIAP2L2 protein expression in normal and HCC tissues were downloaded from HPA (https://www.proteinatlas.org/)^30^ and analyzed to estimate the differences in BAIAP2L2 expression at the protein level. CAMOIP (http://www.camoip.net/) database was used to analyze the distribution of microsatellite instability (MSI) and tumor mutation burden (TMB) in BAIAP2L2 low or high expression cohort.

### Survival analysis

Survival data of 374 HCC patients were obtained from the level 3 HTSeq-FPKM format in the TCGA–LIHC project using the survival package and survminer package for statistical analysis and visualization, respectively. Survival data were acquired for HCC patients in the high and low BAIAP2L2 expression groups (splitting patients by automatically selecting the best cutoff) with different clinical characteristics using the Kaplan‒Meier Plotter database (http://kmplot.com/analysis/index.php?p=service&cancer=pancancer_rnaseq) and visualized using the ggplot2 package.

### Immune infiltration analysis

The relationship between BAIAP2L2 expression and immune cell enrichment scores was investigated using independent-sample t tests. Subsequently, the “GSVA” package and ssGSEA algorithm were used to determine the correlation between BAIAP2L2 expression and immune cell species in patients with HCC in the TCGA and GEO (GSE39791) database. BAIAP2L2 expression in different immune cells was analyzed based on the LIHC_GSE140228_Smartseq2 dataset showing the landscape and dynamics of single immune cells in the TISCH database (http://tisch.comp-genomics.org/gallery/)^31,32^.

### BAIAP2L2 regulates immune-related, cuprotosis-related, differentially expressed, and prognosis-related genes

RNAseq data in level 3 HTSeq-FPKM format were downloaded from the TCGA-LIHC project, and the stat package was used for single gene correlation analysis. A total of 422 BAIAP2L2-related genes were screened against *P* < 0.05 and |cor-Spearman|> 0.4 criteria using the stat package. A total of 1,793 immune-related genes (IRGs) were obtained from the ImmPort database (https://www.immport.org/shared/home). The cuprotosis-related genes were FDX1, LIAS, DLD, LIPT1, DLAT, PDHA1, MTF1, PDHB, GLS, and CDKN2A^33^. Differentially expressed genes regulated by BAIAP2L2 were screened using the DEseq2 package^34^ with *P*_*adj*_ < 0.05 and |log2FC|> 0.35 as the criteria. Statistical analysis of survival data was performed with *P* < 0.05 as the threshold using Cox regression analysis and the survival package. Correlation analysis was performed using the spearman method, and the results were visualized in a co-expression heat map using the ggplot package. The survminer package was applied for visualization and screening of prognosis-related genes in HCC.

### Methylation analysis

The correlation between BAIAP2L2 methylation and immunoinhibitory and immunostimulatory factors was analyzed using the TISIDB database. Then, the relationship between BAIAP2L2 methylation levels was evaluated in HCC and paraneoplastic tissues using the human disease methylation database DiseaseMeth version 2.0 (http://bio-bigdata.hrbmu.edu.cn/diseasemeth/) and methylation analysis of TCGA plates in the UALCAN database (http://ualcan.path.uab.edu/cgi-bin/ualcan-res.pl)^35,36^. Gene visualization panel of the MethSurv database (https://biit.cs.ut.ee/methsurv/) was used to perform a heat map analysis of the correlation between BAIAP2L2 expression and methylation sites. Finally, survival analysis was performed to assess the prognosis of patients with HCC at different methylation sites with the LIHC–TCGA March 2017 dataset in the MethSurv database. We incorporated all the relationships to islands, genomic regions, and CpG sites.

### GO/KEGG enrichment analysis

The org.Hs.eg.db package and ClusterProfiler package^37^ were implemented for ID conversion and GO/KEGG pathway enrichment analysis, respectively. The data (*P*_*adj*_ < 0.05) were visualized and analyzed using the ggplot2 package.

### Protein‒protein interaction (PPI) network

We downloaded the STRING interactions short file of 75 BAIAP2L2 coexpressed genes and prognosis-related genes in HCC using the multiple proteins board of the STRING database (https://cn.string-db.org/)^38^. Then, the top 15 hub genes were selected for network reconstruction with Cytoscape_v3.9.1^39^.

### Drug sensitivity analysis of BAIAP2L2

The transcriptomic and drug sensitivity correlation data (RNA__RNA_seq_composite_expression and DTP_NCI60_ZSCORE) of BAIAP2L2 were downloaded from CellMiner^40^. A Pearson correlation test was conducted to analyze the relationship between BAIAP2L2 expression and drug sensitivity. The impute package and limma package^41^ were used for identifying the relationship between the differential expression of BAIAP2L2 and drug sensitivity with the ggplot2 package and ggpubr package for visualization.

### Cell culture and transfection

Four cell lines, L02(YUCHI BIOLOGY, Shanghai, China), LM3 (CELLCOOK, Guangzhou, China), Huh-7 (CELLCOOK, Guangzhou, China), and Hep-G2 (CELLCOOK, Guangzhou, China), were used in this study. The cells were treated with Dulbecco’s modified Eagle’s medium (DMEM, BasalMedia, China) supplemented with 10% fetal bovine serum (ABW, URU) in a 5% CO_2_ incubator at 37 °C and saturated humidity. Huh-7 and LM3 cells were plated into 6-well plates. The BAIAP2L2 siRNAs (si-1 and si-2) and corresponding scrambled siRNA control (NC) were obtained from GenePharma (Shanghai, China) (Supplemental Table S1). The siRNAs were transfected into cells using Lipofectamine 2000 reagent (Invitrogen, USA).

### Semiquantitative and real-time PCR

Semiquantitative and real-time fluorescence quantification was performed using Transgene SYBR Green dye according to the manufacturer’s protocol. BAIAP2L2 was amplified using the following primers: forward, 5′-GCGGCACTTGAACTCTGACC-3′, and reverse, 5′-GCCACAGCTCAGACATGCAC-3′. GAPDH was used as an endogenous control with the following primers: 5′-GGTCGGAGTCAACGGATTTG-3′ and reverse 5′-GGAAGATGGTGATGGGATTT-3′.

### Western blotting analysis

RIPA lysate buffer (Solarbio, Beijing, China) was used to extract total protein from 4 cell lines grown in logarithmic stage, and protease inhibitors (MCE, Shanghai, China) were added before use. Total protein was quantified by BCA kits (Thermo Fisher Scientific, USA). Then, 10–20 μg protein was separated by 10% SDS‒PAGE gels and transferred to polyvinylidene fluoride (PVDF) membranes. After blocking the membranes with 5% nonfat milk, they were incubated with primary antibodies at room temperature for 2 h or at 4 °C overnight, followed by incubation with secondary antibodies (Thermo Fisher Scientific, USA) at room temperature for 1 h, and then visualized by chemiluminescence reagents (Thermo Fisher Scientific, USA).

### Wound healing assay

Cells were seeded overnight in 6-well plates. After being transfected with siRNA, a straight linear wound was made in each well by using a 10 μL pipette tip. Wound healing images were taken at 0, 12 and 24 h.

### Transwell assay

Huh-7 and LM3 cells were inoculated in the upper chamber of a transwell insert with 8 µm pores (Corning Inc., NY-Corning, USA) with 100 mL serum-free medium, and the lower chamber (with matrix gum/without matrix gum) was filled with 500 mL medium containing 20% FBS for 24 h. Five fields were randomly selected to capture images, and the number of cells passing through the chamber was counted.

## Results

### The BAIAP2L2 expression in HCC

**Figure 1:**
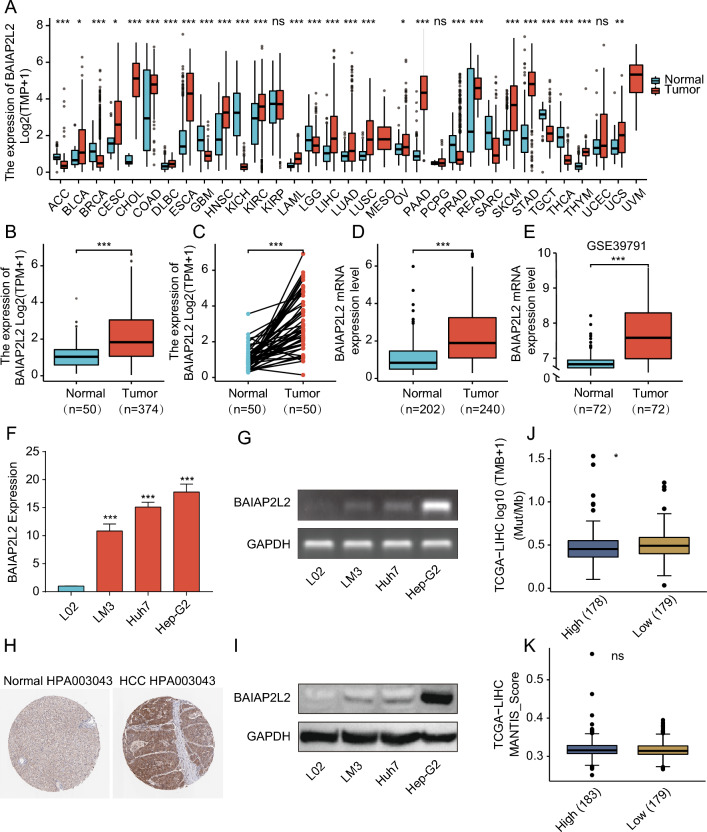
The expression of BAIAP2L2 in HCC and pancancer datasets. (**A**) The mRNA levels of BAIAP2L2 in pancancer TCGA datasets. (**B, C**) The mRNA levels of BAIAP2L2 in HCC and normal tissues from the TCGA database; (**B**) unpaired samples; (**C**) paired samples. (**D, E**) The mRNA expression of BAIAP2L2 in HCC (**D**) from the ICGC and (**E**) GEO (GSE39791) datasets. (**F, G**) The mRNA expression of BAIAP2L2 in L02 and 3 HCC cell lines. (**F**) RT‒qPCR. (**G**) AGE. (**H, I**) BAIAP2L2 protein expression in HCCs from the (H) HPA database and (**I**) western blotting assay. (**J, K**) the distribution of (**J**) TMB and (**K**) MSI in BAIAP2L2 low or high expression cohort. AGE: agarose gel electrophoresis; GEO: Gene Expression Omnibus; HCC: HCC; HPA: Human Protein Atlas; ICGC: International Cancer Genome Consortium; TCGA: The Cancer Genome Atlas; TMB: Tumor Mutation Burden; MSI: Microsatellite Instability. *P < 0.05, **P < 0.01, ***P < 0.001.

The majority of tumors (18/33) in the TCGA dataset had significantly higher BAIAP2L2 expression levels than paraneoplastic tissues (Fig. 1A). BAIAP2L2 expression was significantly upregulated in HCC samples compared to normal samples (*p* < 0.001; Fig. 1B). We also found the same trend in BAIAP2L2 expression in paired HCC samples (*P* < 0.001; Fig. 1C). BAIAP2L2 expression was validated in HCC using the ICGC and GEO datasets (Fig. 1D, E). In addition, semiquantitative PCR and RT‒qPCR confirmed that the expression of BAIAP2L2 was higher in HCC cell lines (LM3, Huh7, and Hep-G2) than in normal hepatocytes (L02) (Fig. 1F, G). Consistent with the mRNA expression data, BAIAP2L2 protein expression was higher in HCC tissues than in paraneoplastic tissues (Fig. 1H). We further validated the level of protein expression in cell lines and obtained the same result (Fig. 1I). High BAIAP2L2 expression had high tumor mutational burden, while microsatellite instability did not differ between high and low BAIAP2L2 expression groups in HCC (Fig. 1J, K). As shown in Supplementary Fig. S1B-I, BAIAP2L2 expression levels were higher in stage T3 than in stage T1. BAIAP2L2 expression was higher in pathologic stage III patients than in stage I patients, and the BAIAP2L2 expression level was higher in the tumor group than in the tumor-free group. BAIAP2L2 expression was higher in women than in men. The BAIAP2L2 expression level was higher in patients aged ≤ 60 years, in the residual tumor group, and in patients with serum AFP levels > 400. The BAIAP2L2 expression level was higher in patients who died than in those who survived. These results strongly demonstrate that BAIAP2L2 is significantly upregulated in HCC at the mRNA and protein levels.

### Prognostic value of BAIAP2L2 in HCC

**Figure 2:**
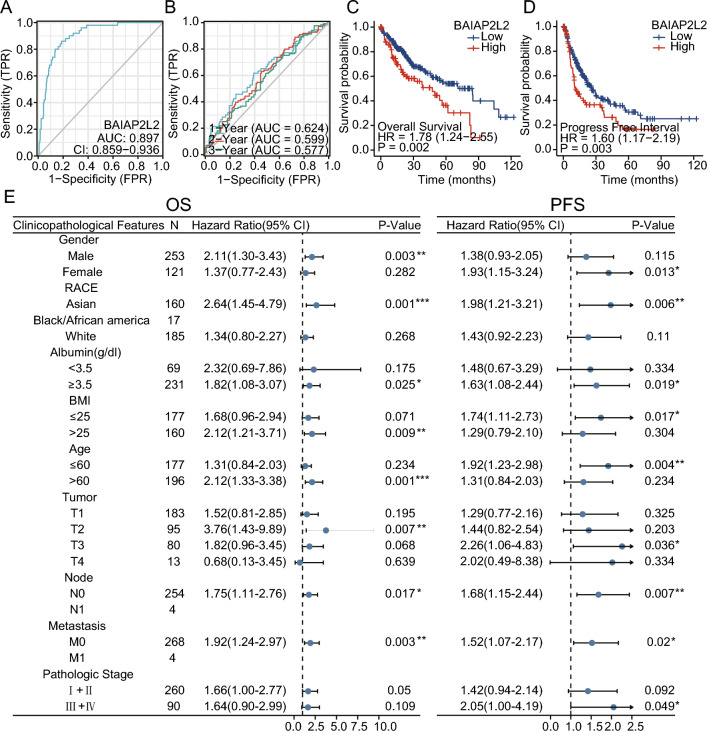
Diagnostic and prognostic values of BAIAP2L2 in HCC. (**A**) The area under the ROC curve for BAIAP2L2 expression in HCC. (**B**) Time-dependent ROC curve analysis of BAIAP2L2 expression in the HCC. **(C, D**) Relationship between BAIAP2L2 expression and the (**C**) OS and (**D**) PFI of HCC patients. (**E**) Forest plot of BAIAP2L2 expression associated with clinicopathological parameters in HCC. ROC: Receiver operator characteristic curve, blue circles represent hazard ratio; PFI: progression-free interval; OS: overall survival. *P < 0.05, **P < 0.01.

The basic information of the 374 HCC patients with level 3 HTSeq data in the TCGA–LIHC dataset is shown in Table 1. ROC curve analysis showed that BAIAP2L2 expression could distinguish tumor tissues from normal tissues in HCC patients (AUC: 0.897, 95% CI 0.859–0.936, Fig. 2A). To further explore the relationship between the prognosis and BAIAP2L2 expression in HCC, we performed a time-dependent ROC curve analysis, which showed that the accuracy of BAIAP2L2 in predicting the prognosis of patients with HCC decreased with increasing time (AUC: 1 year = 0.624; 2 years = 0.599; 3 years = 0.577, Fig. 2B). In addition, survival analysis revealed that a low BAIAP2L2 expression level was associated with better overall survival (OS) (hazard ratio (HR)  = 1.78, 95% confidence interval (CI) 1.24–2.56, *P* = 0.002) and a shorter progression-free interval (PFI) (HR = 1.60, 95% CI 1.17–2.19, *p* = 0.003) (Fig. 2C, D). The subgroup analysis of OS and PFI according to different clinicopathological stages suggested that HCC patients with low BAIAP2L2 expression had better OS and PFI in the Asian, albumin ≥ 3.5 g/dL, N0 and M0 groups. Furthermore, HCC patients with high BAIAP2L2 expression had worse OS in the male, BMI > 25, age > 60 and T2 groups. The same trend was observed for PFI in women, BMI ≤ 25, age ≤ 60, stage T3 and pathological stage 3 and 4 groups (Fig. 2E).

**Table 1:** Correlation between BAIAP2L2 expression and the clinicopathological features of the HCC cases from TCGA.

Characteristic	Low expression of BAIAP2L2	High expression of BAIAP2L2	*P* value
n	187	187	
T stage, n (%)			0.150
T1	100 (27%)	83 (22.4%)	
T2	47 (12.7%)	48 (12.9%)	
T3	34 (9.2%)	46 (12.4%)	
T4	4 (1.1%)	9 (2.4%)	
N stage, n (%)			0.368
N0	129 (50%)	125 (48.4%)	
N1	1 (0.4%)	3 (1.2%)	
M stage, n (%)			1.000
M0	133 (48.9%)	135 (49.6%)	
M1	2 (0.7%)	2 (0.7%)	
Gender, n (%)			0.002**
Female	46 (12.3%)	75 (20.1%)	
Male	141 (37.7%)	112 (29.9%)	
Age, n (%)			0.034*
≤ 60	78 (20.9%)	99 (26.5%)	
> 60	109 (29.2%)	87 (23.3%)	
Race, n (%)			0.074
Asian	77 (21.3%)	83 (22.9%)	
Black or African American	13 (3.6%)	4 (1.1%)	
White	89 (24.6%)	96 (26.5%)	
Child–Pugh grade, n (%)			0.648
A	115 (47.7%)	104 (43.2%)	
B	12 (5%)	9 (3.7%)	
C	0 (0%)	1 (0.4%)	
AFP (ng/ml), n (%)			
≤ 400	122 (43.6%)	93 (33.2%)	0.008*
> 400	24 (8.6%)	41 (14.6%)	

Significant values are in [bold].

### Relationship of BAIAP2L2 expression and immune infiltration in HCC

**Figure 3:**
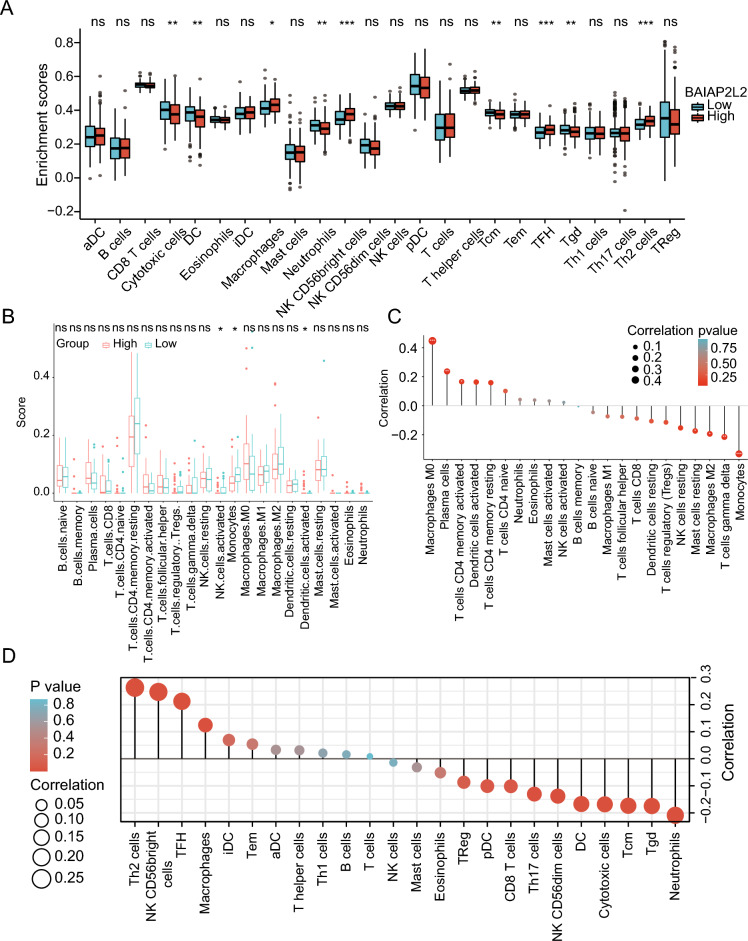
Correlation analysis of BAIAP2L2 expression and immune infiltration in HCC. (**A, B**) Differential distribution of immune cells between samples with high and low BAIAP2L2 expression in (**A**) TCGA and (**B**) GEO database. (**C,D**) The relationship between BAIAP2L2 expression and the levels of tumor-infiltrating cell in (**C**) GEO and (**D**) TCGA database. *P < 0.05, **P < 0.01, ***P < 0.001.

We compared the enrichment scores of immune cells from the TCGA database between the high and low BAIAP2L2 expression groups to determine whether BAIAP2L2 expression was correlated with the level of immune infiltration in HCC. The results showed that the immune infiltration level of dendritic cells (DCs) and DCs-activated were higher in the high BAIAP2L2 expression group than in the low BAIAP2L2 expression group (Fig. 3A, B). However, the correlation was not consistent between the expression levels of BAIAP2L2 and immune cells in the TCGA and GEO databases (Fig. 3C, D). In addition, the expression of BAIAP2L2 in immune cells indicated that BAIAP2L2 was mainly expressed in DCs, CD4Tconv cells, CD8Tex cells, NK cells, and monocytes/macrophages according to the LIHC_GSE140228_Smartseq2 dataset of the TISCH database (Supplementary Fig. S2A–C). In summary, these results suggest that BAIAP2L2 may be closely associated with immune infiltration during the progression of HCC.

### Prognosis analysis of BAIAP2L2-regulating IRGs in HCC

**Figure 4:**
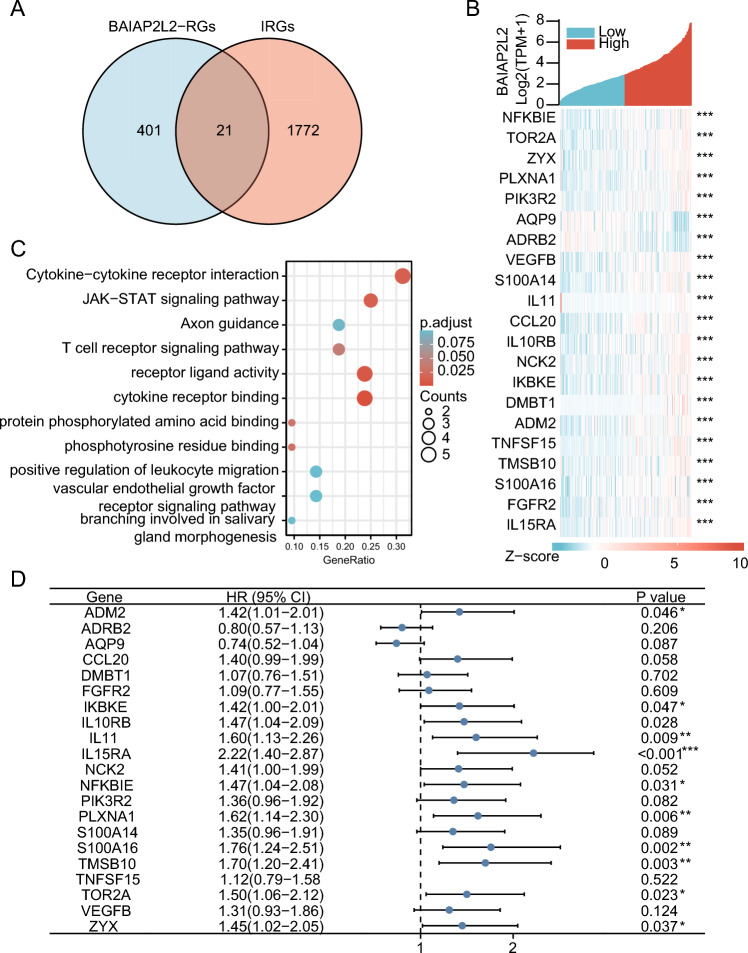
Enrichment analysis of BAIAP2L2-RGs and IRGs. (**A**) Venn diagram of BAIAP2L2-RGs and IRGs. (**B**) Heatmap of coexpression analysis of IRGs regulated by BAIAP2L2 and BAIAP2L2 using R Software (Version 4.2.1 https://cran.r-project.org/src/base/R-4/). (**C**) GO/KEGG enrichment analysis of overlapping BAIAP2L2-RGs and IRGs. (**D**) Forest map of overlapping BAIAP2L2-RGs and IRGs. BAIAP2L2-RGs: BAIAP2L2-related genes; IRGs: immune-related genes; *P < 0.05, **P < 0.01, ***P < 0.001.

There is a consensus that the development of tumors causes changes in the immune microenvironment, influenced by the alteration of various immune cells and IRGs^42^. We screened 21 BAIAP2L2-related genes from 1793 IRGs (Fig. 4A) and subsequently performed a coexpression analysis of these 21 genes with BAIAP2L2 to assess the relationship between BAIAP2L2 and immune regulation in HCC. AQP9 and ADRB2 expression was negatively correlated with BAIAP2L2 expression, whereas the expression of remaining molecules was positively correlated with BAIAP2L2 expression (Fig. 4B). GO/KEGG enrichment analysis of 21 IRGs showed enrichment in cytokine‒cytokine receptor interaction, the JAK-STATA signaling pathway, axon guidance, and the T-cell receptor signaling pathway (Fig. 4C). Survival analysis, ADM2, IKBKE, IKBKE, IL11, IL15RA, PLXNA1, S100A16, TMSB10, and TOR2A were associated with poor prognosis in HCC patients (Fig. 4D).

### Methylation analysis

**Figure 5:**
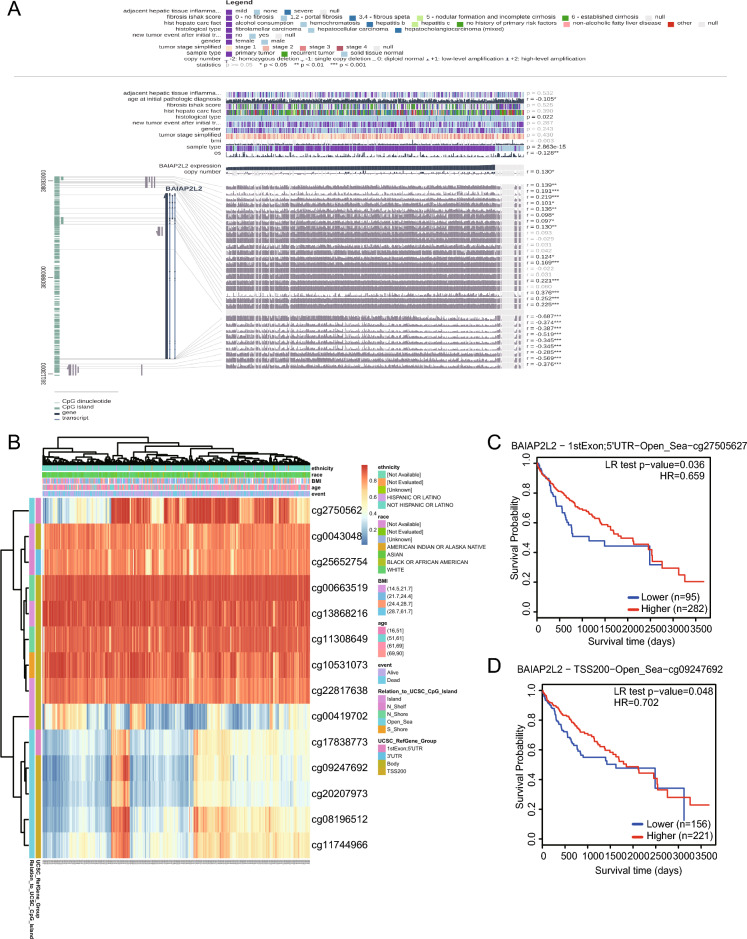
Methylation analysis of BAIAP2L2 in HCC. (**A**) Summary of BAIAP2L2 methylation in HCC. (**B**) Heatmap of BAIAP2L2 methylation sites in HCC using MethSurv database (https://biit.cs.ut.ee/methsurv/). (**C**,**D**) Survival curves based on two methylation sites affecting survival. *P < 0.05, **P < 0.01, ***P < 0.001.

Methylation is a common epigenetic modification. Promotion of methylation usually inhibits the transcriptional processes of genes to influence gene expression. The DNA methylation level of BAIAP2L2 was significantly reduced in HCC tissues compared with that in normal samples according to the DiseaseMeth version 2.0 and UALCAN databases (Supplementary Fig. S3A, B). Furthermore, the methylation level of BAIAP2L2 was lower in women with advanced and high-grade tumors than in those with less aggressive tumors (Supplementary Fig. 3C–E). Interestingly, we identified nine methylation sites (cg27505627, cg17838773, cg20207973, cg08196512, cg11744966, cg09247692, cg11461302, cg15944459, and cg21007971) in the DNA sequence that were negatively correlated with BAIAP2L2 expression levels (Fig. 5A, B). Furthermore, BAIAP2L2 hypermethylation at the cg27505627 (HR = 0.659, *P* = 0.036) and cg09247692 (HR = 0.702, *P* = 0.048) sites in HCC (Fig. 5C, D) was associated with a poor prognosis. Recently, it was reported that the immune status of an organism may affect the level of methylation^43^. According to the TISIDB database, BAIAP2L2 methylation status was positively correlated with the expression level of immunosuppressants, including BTLA (rho = 0.126, *P* = 0.0145), CD244 (rho = 0.162, *P* = 0.00168), CD96 (rho = 0.128, *P* = 0.0138), and KDR (rho = 0.247, *P* = 1.53e^−06^) (Supplementary Fig. S4A–D), whereas it was negatively correlated with the levels of immune activators HHLA2 (rho = -0.221, *P* = 1.75e^−05^) and ULBP1 (rho = − 0.226, *P* = 1.07e^−05^) (Supplementary Fig. S4E, F). These results indicated that the methylation status of BAIAP2L2 might be positively correlated with immunosuppression status, and thus the methylation level of BAIAP2L2 could serve as a potential prognostic biomarker and might play a critical role in the progression of HCC.

### Prognosis analysis of BAIAP2L2-regulating cuprotosis-related genes in HCC

**Figure 6:**
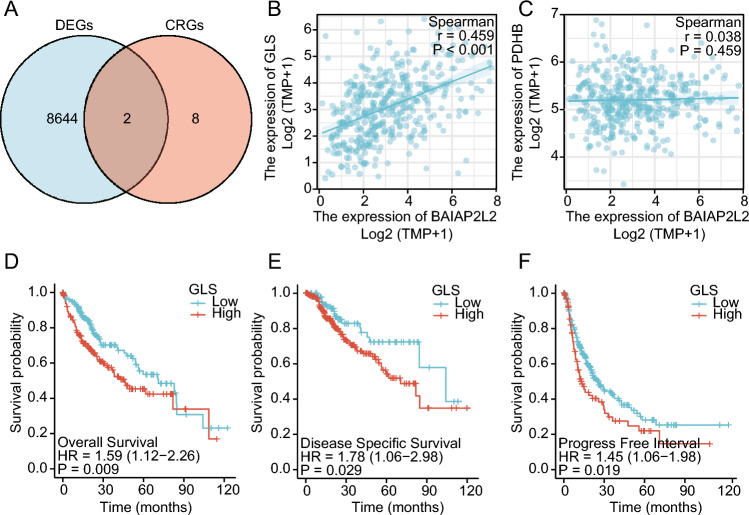
Correlation analysis of BAIAP2L2 and cuproptosis-related genes. (**A**) Venn diagram of BAIAP2L2-related DEGs and CRGs. (**B**, **C**) Scatter plot of the correlation analysis between BAIAP2L2 and overlapping genes. (**D–F**) The prognostic values of GLS (**D**) in terms of OS (**E**), DSS, and (**F**) PFI. CRGs: Cuproptosis-related genes; DSS: disease-specific survival; DEGs: differentially expressed genes.

Cuproptosis is a novel cell death pathway^33^. To investigate whether BAIAP2L2 is implicated in cuprotosis, we screened two BAIAP2L2-regulated cuprotosis-related genes (GLS and PDHB) by taking the intersections of 8,646 BAIAP2L2-regulated DEGs and 10 cuproptosis-related genes in HCC (Fig. 6A). GLS expression was positively correlated with BAIAP2L2 expression (r = 0.459, *P* < 0.001, Fig. 6B), whereas PDHB expression was not related to BAIAP2L2 expression (r = 0.038, *P* = 0.459, Fig. 6C) in HCCs. Further survival analysis demonstrated that low GLS expression was a favorable factor in terms of the survival of HCC patients (OS: HR = 1.59, 95% CI 1.12–2.26, *P* = 0.009; DSS: HR = 1.78, 95% CI 1.06–2.98, *P* = 0.029; PFI: HR = 1.45, 95% CI 1.06–1.98, *P* = 0.019) (Fig. 6D–F). Therefore, we speculated that BAIAP2L2 might affect HCC prognosis by modulating cuprotosis-related genes.

### Coexpression analysis

**Figure 7:**
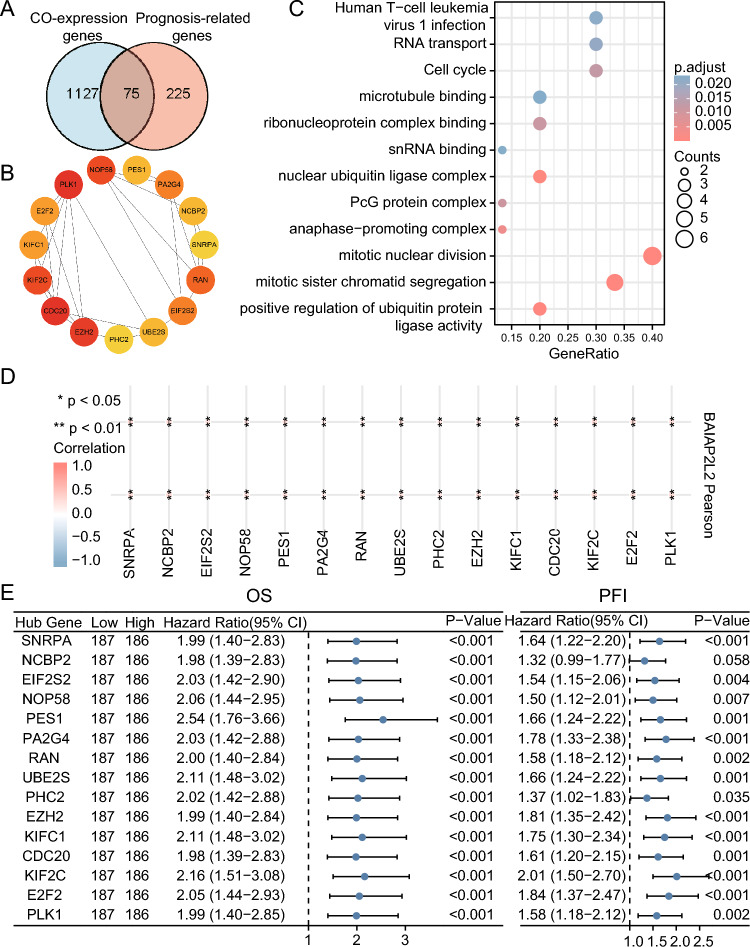
Hub gene analysis. (**A**) Venn diagram of BAIAP2L2 coexpressed genes and prognostic genes of HCC. (**B**) The interaction network of the top 15 hub genes. (**C**) GO/KEGG analysis of the top 15 hub genes. (**D**) Heatmap of the correlation between 15 hub genes and BAIAP2L2 using R Software (Version 4.2.1 https://cran.r-project.org/src/base/R-4/). (**E**) Forest map of OS and FPI of 15 hub genes in HCC. *P < 0.05, **P < 0.01, ***P < 0.001.

We identified 75 BAIAP2L2-coexpressed genes from 300 prognosis-related genes in HCC (Fig. 7A). The first 15 hub genes of the 75 genes were identified using cytoHubba (Fig. 7B). GO/KEGG analysis of these 15 hub genes showed enrichment of biological processes, including human T-cell leukemia virus 1 infection, RNA transport, and the cell cycle (Fig. 7C). These 15 hub genes were positively correlated with BAIAP2L2 expression in HCC according to correlation analysis (Fig. 7D). Except for NCBP2 (HR = 1.32, 95% CI 0.99–1.77, *P* = 0.058), high expression of the remaining 14 hub genes was associated with poor OS in HCC (Fig. 7E). Hence, BAIAP2L2 might affect the prognosis of HCC by interacting with coexpressed genes.

### Drug sensitivity analysis

**Figure 8:**
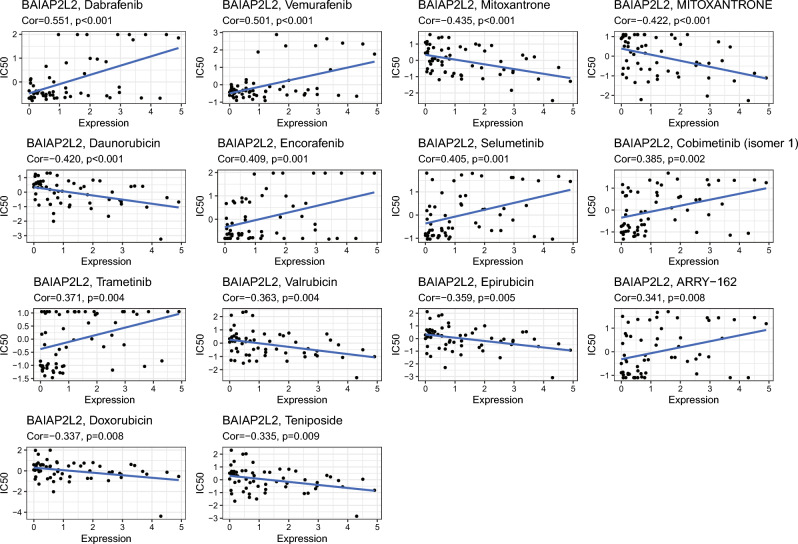
Correlation analysis of BAIAP2L2 expression and antitumor drug sensitivity.

Systemic drug therapy was critical for HCC. We downloaded gene expression and drug sensitivity data from CellMiner and screened FDA-approved drugs, which were analyzed the correlation coefficients between BAIAP2L2 expression and drug sensitivity to investigate the relationship between BAIAP2L2 and antitumor drugs. The results showed that high BAIAP2L2 expression was associated with better sensitivity to 7 drugs, including dabrafenib and vemurafenib. and increased resistance to 6 drugs, including mitoxantrone and daunorubicin (Fig. 8). These results implied that sensitivity to certain drugs could be determined based on the expression of BAIAP2L2.

### The effects of BAIAP2L2 on migration and invasion of HCC cells

**Figure 9:**
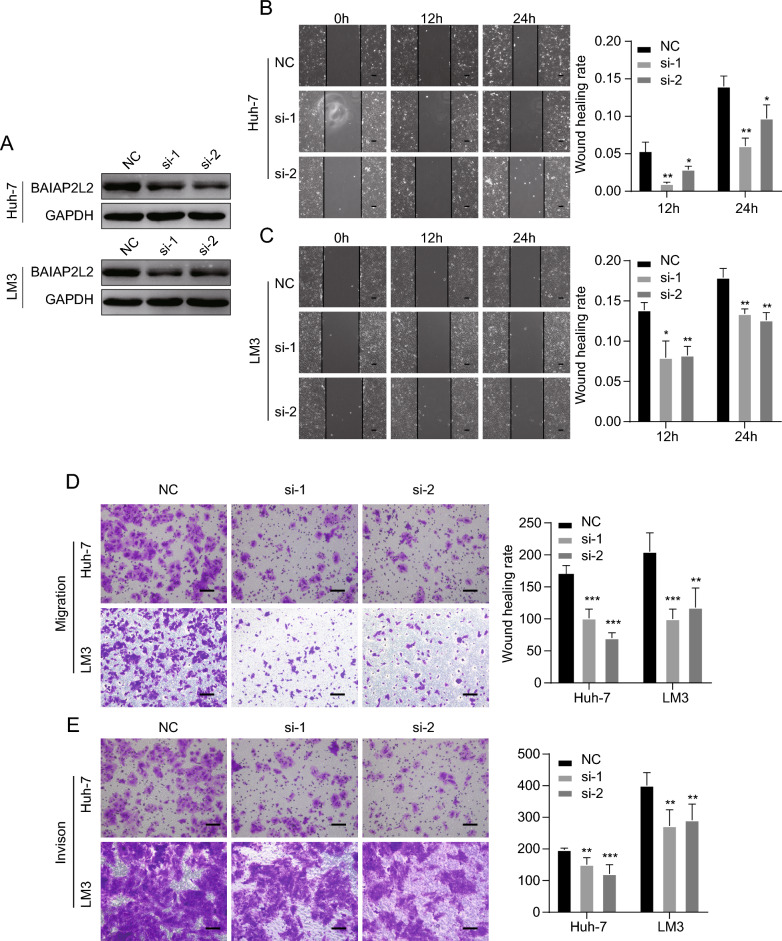
Down-regulation of BAIAP2L2 inhibited migration and invasion of HCC cells. (**A**) Western blotting showed that the downregulation of BAIAP2L2 was successful. (**B, C**) Cell migration was measured using wound scratch assay. (**D, E**) Cell migration and invasion were measured using transwell assay. Scale bar, 500 μm *P < 0.05, **P < 0.01, ***P < 0.001.

To determine the roles of BAIAP2L2 in the progression of HCC cells. BAIAP2L2 was knocked down in HCC cells using si-RNA transfection. Knockdown efficiency was verified by western blotting (Fig. 9A). In the wound healing assays, siRNA-induced downregulation of BAIAP2L2 led to a decrease in the wound healing rate due to the significantly decreased cell migration ability in both Huh-7 and LM3 cells (Fig. 9B, C). In the transwell assays, the decrease in BAIAP2L2 expression gave rise to a significant decrease in the number of cells migrating and invading through the chamber in both Huh-7 and LM3 cells (Fig. 9D, E). Taken together, the data indicate that BAIAP2L2 may be required for cancer cell migration and invasion in HCC.

## Discussion

HCC remains a prevalent tumor that threatens human health. The main causes included hepatitis B and hepatitis C infection, alcohol addiction, and intake of toxic chemicals, such as aflatoxins. Unfortunately, as with other solid tumors. HCC is commonly diagnosed at an advanced stage, with limited treatment options and poor patient prognosis. Previous studies have illustrated that BAI-related proteins maintain their regulatory role in inflammation and phagocytosis in the process of tumorigenesis^44^. BAIAP2L2 is an integral member of the I-BAR protein family and has been identified as a possible biomarker for certain diseases. BAIAP2L2 has been reported to be a marker for lung cancer^12^. However, current research on BAIAP2L2 in HCC remains limited. BAIAP2L2, a junction protein, is a 529 amino acid protein containing the SH3 and IMD (IRSp53/MIM) domains that enable BAIAP2L2 to bind actin filaments and membranes to interact with Rac GTPase. Recently, various studies have suggested that BAIAP2L2 may be involved in tumor progression; however, comprehensive bioinformatic analysis of BAIAP2L2 in HCC is rare.

In this study, using the TCGA database, the expression of BAIAP2L2 was found to be significantly higher in HCC tissues than in normal tissues. The findings were externally validated using qRT‒PCR, ICGC, and GEO datasets. Moreover, using the HPA database, we found that BAIAP2L2 protein was highly expressed in HCC. Previous studies have revealed that BAIAP2L2 has predictive value in prostate and lung cancers. The area under the ROC curve indicated that BAIAP2L2 had promising value in predicting the occurrence of HCC (AUC = 0.897). BAIAP2L2 has been found to be a biomarker for HCC recurrence. Furthermore, by survival analysis, we found that high expression of BAIAP2L2 was associated with poor OS and a short PFI in HCC patients, suggesting that reducing the expression of BAIAP2L2 may improve the prognosis of patients with HCC.

Immunosuppression is frequently observed at tumor sites. Transformed malignant cells rarely resist an attack by the immune system, but those that survive alter the phenotype to reduce immunogenicity^45^. Our study demonstrated a higher degree of Th2 cell immune infiltration in the high BAIAP2L2 expression group than in the low BAIAP2L2 expression group in HCC patients. Some studies have confirmed that Th2 cells expressing IL-13, IL-5, and IL-4 recruit M2-like tumor-associated macrophages and contribute to tumor angiogenesis by activating STAT-6^46–49^. Importantly, our enrichment analysis of IRGs regulated by BAIAP2L2 showed enrichment in the JAK-STAT signaling pathway. Li et al. demonstrated that immunogenic death of HCC cells could be induced by targeting STAT3 inhibition through glycolysis. Hence, it was reasonable to assume that BAIAP2L2 boosted Th2 cell infiltration in HCC, which was detrimental to the prognosis of patients with HCC. In addition, defective DC recruitment can result in impaired antitumor immunity^50^. DC activation can enhance macrophage recognition and phagocytosis in HCC cells^51^. We found that the expression of BAIAP2L2 was negatively correlated with the number of DCs, so we inferred that BAIAP2L2 might restrain the activation of DCs and thereby enable the proliferation of cancer cells to further promote the progression of HCC. Overall, these results indicate that BAIAP2L2 may be closely associated with immune infiltration during HCC progression.

Epigenetics has been documented as a novel method to regulate tumors reversibly. DNA methylation is one of the most common epigenetic mechanisms in cancer. In general, methylation of gene promoter regions leads to transcriptional repression, whereas methylation of gene bodies promotes gene expression^52^. It has been shown that DNA methylation defects are closely related to HCC^53^. However, the role of BAIAP2L2 as an oncogene in HCC is unknown. We found that BAIAP2L2 exhibited promoter hypermethylation in HCCs, which may increase the transcriptional activity of BAIAP2L2 and render BAIAP2L2 highly expressed in HCCs. We further investigated the relationship between specific methylation sites of BAIAP2L2 and prognosis in HCC and found that the methylation levels of two sites, cg27505627 (*p* = 0.036) and cg09247692 (*p* = 0.048), affected the prognosis of HCC patients. Therefore, we presumed that the oncogenic effect of BAIAP2L2 was related to methylation of the promoter region.

An ideal solution in oncology research is to effectively kill tumor cells while keeping healthy cells intact. Eleven types of cell death, including apoptosis, heat stress-induced death, autophagy, iron death, and cell-in-cell structure, have been identified, and their mechanisms differ in tumor cells. Golub et al. demonstrated that cuprotosis, a copper ion carrier-induced death dependent on the accumulation of intracellular copper, was a novel cell death pathway^33^. It was unclear whether BAIAP2L2 is associated with cuproptosis in HCC patients. GLS can negatively regulate cuprotosis^26^. We found that BAIAP2L2 was correlated with the expression of cuprotosis-related genes (GLS). In addition, survival analysis revealed that HCC patients with high GLS expression had worse OS, DFS, and PFI than those with low GLS expression. Glutamine catabolism is a central metabolic process that promotes the proliferation of cancer cells, including HCC cells^54^. It has been shown that knockdown of GLS inhibits the proliferation of HCC^55^. Furthermore, GLS activity, but not GLS1 or GLS2 expression, was a critical factor in activating mTORC1 and promoting HCC development^56^. Our correlation analysis showed that BAIAP2L2 was significantly and positively correlated with GLS; therefore, it was reasonable to speculate that BAIAP2L2 and GLS exert synergistic effects to promote the progression of HCC.

We analyzed 15 hub genes (SNRPA, NCBP2, EIF2S2, NOP58, PES1, PA2G4, RAN, UBE2S, PHC2, EZH2, KIFC1, CDC20, KIF2C, E2F2, and PLK1) coexpressed with BAIAP2L2 in HCC to investigate the potential function of BAIAP2L2 in HCC. Survival analysis revealed that all 15 genes were associated with OS and PFI in HCC. It has been demonstrated that PES1 and KIF2C can promote the proliferation of HCC^57–59^. Furthermore, phosphorylation of PLK1 can induce G2/M cell cycle arrest in HCC^60^. Zhang et al. found that UBE2S accelerated HCC development by enhancing the ubiquitination of p27^61^. Moreover, UBE2S enhanced the ubiquitination of p53 and exerted oncogenic activity in HCC^62^. Thus, we surmised that BAIAP2L2 might influence the progression and prognosis of HCC by interacting with hub genes to regulate the cell cycle and ubiquitination process.

Systemic drug therapy is an important treatment modality for HCC. Following the approval of sorafenib as first-line systemic therapy in patients with advanced HCC, lenvatinib, regorafenib, cabozantinib, ramucirumab, immune checkpoint inhibitors, and other drugs have been successively administered for systemic drug therapy of HCC^63–66^. HCC usually presents an immune “cold” state, which protects cancer cells from tumor-infiltrating lymphocytes, resulting in poor immunotherapy responses^67^. Therefore, it is imperative to identify new drugs and the mechanisms of resistance to existing drugs. We performed a drug sensitivity analysis of BAIAP2L2 and found that BAIAP2L2 expression was positively correlated with sensitivity (dabrafenib, vemurafenib, encorafenib, selumetinib, cobimetinib, trametinib, and ARRY-162) and resistance (mitoxantrone, daunorubicin, valrubicin, epirubicin, doxorubicin, and teniposide).

## Conclusions

In this study, we demonstrated that BAIAP2L2 was closely associated with HCC. Moreover, BAIAP2L2 overexpression in HCC was verified using TCGA, ICGC, and GEO databases. We further determined the correlation of BAIAP2L2 expression with prognosis, immune infiltration, methylation, cuproptosis, and drug sensitivity in HCC and assessed its coexpressed genes. Moreover, knockdown of BAIAP2L2 can affect the migration and invasion of HCC cells. Our work revealed the role of BAIAP2L2 in the progression of HCC, especially in the immune response, tumor microenvironment, and drug resistance, indicating that it could be crucial for developing tailored cancer therapies.

## Supplementary Information

Supplementary Figure S1.

Supplementary Figure S2.

Supplementary Figure S3.

Supplementary Figure S4.

Supplementary Table S1.

Supplementary Figures.

## Data Availability

All of the data involved in this study are available in the public databases (HPA (https://www.proteinatlas.org/), Kaplan–Meier Plotter database (http://kmplot.com/analysis/index.php?p=service&cancer=pancancer_rnaseq), TISCH database (http://tisch.comp-genomics.org/gallery/), ImmPort database (https://www.immport.org/shared/home), DiseaseMeth version 2.0 (http://bio-bigdata.hrbmu.edu.cn/diseasemeth/), UALCAN database (http://ualcan.path.uab.edu/cgi-bin/ualcan-res.pl), MethSurv database (https://biit.cs.ut.ee/methsurv/), STRING database (https://cn.string-db.org/), GEO (GSE39791) and ICGC (LC-RIKEN, JP)).
