# Supplementary material for: BAIAP2L2 is a novel prognostic biomarker related to migration and invasion of HCC and associated with cuprotosis
Source: Sci Rep. 2023 May 29;13:8692. doi: 10.1038/s41598-023-35420-0 (PMC10227027; doi:10.1038/s41598-023-35420-0)
Supplement: Supplementary file 5 — Supplementary Table S1. [file 41598_2023_35420_MOESM5_ESM.docx]

Supplementary table 1. The sequence of BAPAP2L2 siRNAs

| **siRNA** | **Sequence (5’ to 3’)** |
| --- | --- |
| BAIAP2L2_si-1 Sence: | GCAUCAUGGAGCAGUUUAATT |
| Anti-sence: | UUAAACUGCUCCAUGAUGCTT |
| BAIAP2L2_si-2 Sence: | Sence: GACGUCAAGAAACUGAUGUTT |
| Anti-sence: | Anti-sence: ACAUCAGUUUCUUGACGUCTT |
| NC Sence: | Sense: UUCUCCGAACGUGUCACGUTT |
| Anti-sence: | Anti-Sense: ACGUGACACGUUCGGAGAATT |
